# Early Pro-Inflammatory Remodeling of HDL Proteome in a Model of Diet-Induced Obesity: ^2^H_2_O-Metabolic Labeling-Based Kinetic Approach

**DOI:** 10.3390/ijms21207472

**Published:** 2020-10-10

**Authors:** Prabodh Sadana, Li Lin, Mirjavid Aghayev, Serguei Ilchenko, Takhar Kasumov

**Affiliations:** 1 Department of Pharmacy Practice, College of Pharmacy, Northeast Ohio Medical University, Rootstown, OH 44272, USA; 2 Department of Pharmaceutical Sciences, College of Pharmacy, Northeast Ohio Medical University, Rootstown, OH 44272, USA; llin@neomed.edu (L.L.); maghayev@neomed.edu (M.A.); silchenko@neomed.edu (S.I.)

**Keywords:** diet-induced obesity, high-fat diet, high-density lipoprotein, acute-phase proteins, inflammation, proteome dynamics, dyslipidemia, NAFLD, insulin resistance

## Abstract

Mice fed a high-fat diet for 12 weeks or longer develop hyperglycemia, insulin resistance, dyslipidemia, and fatty liver. Additionally, a high-fat diet induces inflammation that remodels and affects the anti-inflammatory and antiatherogenic property of the high-density lipoprotein (HDL). However, the precise time course of metabolic disease progression and HDL remodeling remains unclear. Short-term (four weeks) high-fat feeding (60% fat calories) was performed in wild-type male C57BL/6J mice to gain insights into the early metabolic disease processes in conjunction with a HDL proteome dynamics analysis using a heavy water metabolic labeling approach. The high-fat diet-fed mice developed hyperglycemia, impaired glucose tolerance, hypercholesterolemia without hypertriglyceridemia or hepatic steatosis. A plasma HDL proteome dynamics analysis revealed increased turnover rates (and reduced half-lives) of several acute-phase response proteins involved in innate immunity, including complement C3 (12.77 ± 0.81 vs. 9.98 ± 1.20 h, *p* < 0.005), complement factor B (12.71 ± 1.01 vs. 10.85 ± 1.04 h, *p* < 0.05), complement Factor H (19.60 ± 1.84 vs. 16.80 ± 1.58 h, *p* < 0.05), and complement factor I (25.25 ± 1.29 vs. 19.88 ± 1.50 h, *p* < 0.005). Our findings suggest that an early immune response-induced inflammatory remodeling of the plasma HDL proteome precedes the diet-induced steatosis and dyslipidemia.

## 1. Introduction

It is common for diet-induced metabolic disturbances to manifest as a spectrum of conditions including adiposity, hyperinsulinemia, hyperglycemia, hepatic steatosis, and dyslipidemia [1]. The downstream cardiovascular sequelae of these metabolic disorders, including atherosclerosis, cause significant human morbidity and mortality [2]. The growing epidemic of these cardiometabolic conditions has necessitated an urgent need for a thorough understanding of the development and progression of these diseases. Furthermore, the chronic nature of these conditions and the fact that they often go undetected for several years has emphasized the need for the early indicators of metabolic disturbances [3,4].

The convergence of these multiple diet-induced metabolic disturbances is a significant clinical challenge. Among the animal models used to replicate this human clinical syndrome is high-fat diet (HFD) feeding in mice [5]. The pathophysiological changes produced due to caloric overload associated with high-fat intake mimics the clinical metabolic disease with high fidelity. This model of HFD-induced obesity in mice is marked by hyperinsulinemia, hyperglycemia, non-alcoholic fatty liver disease (NAFLD), and a dysregulated lipoprotein profile—high low-density lipoprotein (LDL)-cholesterol, decreased high-density lipoprotein (HDL)-cholesterol (HDL-C), and hypertriglyceridemia. In addition to the nutritional disbalance, dietary fat intake is also known to result in underlying “low-grade systemic inflammation”. The role of systemic inflammation in the pathogenesis of metabolic diseases, including obesity-associated type 2 diabetes mellitus, NAFLD, and atherosclerotic cardiovascular disease (CVD) is now widely recognized [6,7]. Additionally, the serum levels of HDL are inversely related to CVD risk. However, not merely the levels of HDL, but the composition of HDL particles ultimately determines the CVD risk. Recently more than 100 HDL-associated proteins with distinct functions have been characterized [8]. Alterations in HDL proteome composition can compromise HDL functions and increase CVD risk. Inflammation can profoundly remodel the HDL proteome composition and inhibit its antioxidant, reverse cholesterol transport, and other atheroprotective properties [9,10]. 

Various strains of mice (C57BL/6J, ob/ob, AKR/J, BALB/c) and rats (Sprague–Dawley, Wistar, Zucker, Lewis, Osborne) have shown a varying degree of susceptibility for the development of diet-induced obesity [11,12,13,14]. A commonly used HFD study design is feeding male wild-type C57BL/6J mice an HFD for 12–16 weeks. At the completion of this feeding regimen, mice exhibit the full spectrum of metabolic disorders discussed above [5]. The animal models generated using these relatively long-term feeding studies have significant application in the study of various interventions, including genetic or pharmacological ones [15]; however, they have limited utility in the elucidation of early steps involved in disease manifestation and progression. While there is evidence for insulin resistance even as early as three days after the onset of HFD feeding [16], the timescale of development of early inflammatory signature remains unknown. In particular, the effects of short-term duration of HFD feeding (e.g., four weeks) on metabolic parameters and the underlying pathophysiological processes have not been studied in detail. A previous report has suggested the impairment of glucose tolerance during HFD feeding occurs over two distinct phases—an early phase over the first week of feeding characterized by an acute-phase response and a delayed phase between 12–16 weeks of feeding characterized by inflammation in the adipose tissue and skeletal muscle [17]. Other studies have assessed the time-dependent progressive changes in the metabolic parameters upon HFD feeding [18]; however, the role of the acute-phase response in the HDL proteome composition in short-term HFD feeding has not been evaluated. 

In the early stages of metabolic diseases, the compensatory mechanisms protect against the full-blown metabolic disturbances associated with the changes in static levels of metabolites and proteins. In contrast to static measurements representing the net results of synthesis and degradation, the kinetic analysis of circulatory metabolites and proteins can detect early changes in physiological homeostasis [19]. In this study, we have examined the extent of metabolic disturbances at four weeks after the initiation of HFD in male mice using biochemical and stable isotope-based kinetic analyses. We specifically focused on HDL-associated proteins as they are sensitive to diet-induced inflammation and are involved in reverse cholesterol transport, anti-inflammatory, antioxidant, and antithrombotic functions of HDL that protect against atherosclerosis [20].

## 2. Results

**Figure 1:**
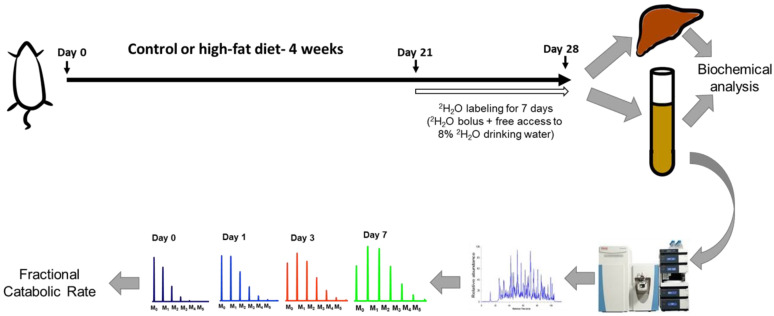
A schematic of the study design. Wild-type C57BL/6J male mice were fed a high-fat diet (HFD) or control diet for four weeks. During the last week of feeding, heavy water was administered to determine the turnover rates of high-density lipoprotein (HDL) proteome. The HDL proteins were isolated, followed by their tryptic digestion and analysis by tandem mass spectrometry. The turnover rate constants of proteins were determined based on their time course of ^2^H-labeling. Metabolic changes were evaluated using biochemical analyses of liver and plasma samples.

The present study used a combination of biological and analytical methods to detail the early metabolic perturbations, including those involved in lipid and glucose homeostasis, in an HFD-induced obesity model. Additionally, we analyzed the plasma HDL proteome dynamics to gain unique insights into concurrent changes in HDL proteome composition using a heavy water-based (^2^H_2_O, a non-radioactive isotope) metabolic labeling approach [21]. This method is based on the rationale that a loading dose of ^2^H_2_O rapidly labels amino acids prior to their incorporation into newly made proteins. The rate of labeled amino acids incorporation into the specific protein depends on the rate of synthesis of the protein. Tryptic peptides are analyzed by high-resolution mass spectrometry and protein turnover rates are assessed based on ^2^H enrichment of peptides (Figure 1) [22]. We have previously applied this method to a mouse model of NAFLD and demonstrated that HFD results in an increased degradation of plasma and hepatic mitochondrial proteins [23,24,25]. 

### 2.1. HFD Induces Partial Perturbation of Metabolic Profile at Four Weeks of Feeding

Since the metabolic disturbances precede the disease onset, the present study was designed with the deliberate focus on identifying early events in metabolic diseases, including NAFLD. Notably, the literature reported that the lengths of “short-term” diet feeding are inconsistent with durations ranging from three days to eight weeks. In the present study, the four-week HFD feeding duration is sufficiently long enough to induce pathophysiological changes that initiate the development of metabolic disease without establishing the complete disease phenotype.

**Figure 2:**
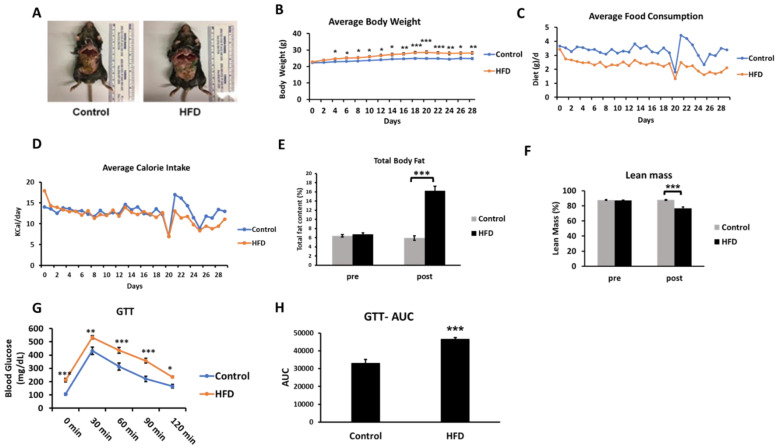
Effects of an HFD in wild-type C57BL/6J male mice at four weeks of feeding (n = 6–7/group). **A**, Representative images of mice fed either the control or HFD. **B**, The change in average body weight over the duration of the study in the control and HFD-fed mice. **C** and **D**, The average daily food consumption and calorie intake in the control and HFD-fed mice. **E** and **F**, Analysis of total fat content and lean mass percentage in mice recorded at the start of the study (pre) and on the day of sacrifice (post) using EchoMRI body composition analyzer. **G** and **H**, The time course and the area under the curve (AUC) of the glucose tolerance test (GTT) in the mice after intraperitoneal injection of glucose following an eight-hour fast. Results are mean ± SEM. Statistical significance, * indicates *p* < 0.05, ** indicates *p* < 0.01, and *** indicates *p* < 0.005 compared to control.

The HFD consumption for four weeks led to an obesogenic effect with mice showing a generally increased adiposity and increased body weight (Figure 2A,B). While the HFD group showed lower average daily food consumption (in grams) (Figure 2C), the two groups were evenly matched in respect to kilocalories consumed each day (Figure 2D). A body composition analysis revealed a significant increase in total body fat in the HFD group (fat content (%), 5.95 ± 0.27 vs. 16.22 ± 0.32, control vs. HFD, respectively) (Figure 2E). A statistically significant decrease in lean mass (%) was observed in the HFD group (76.73 ± 1.58) over the control group (87.76 ± 0.65) (Figure 2F). The HFD mice showed basal fasting hyperglycemia (Figure 2G) and impaired glucose tolerance assessed in the third week of the feeding protocol. The increased area under the curve in the glucose tolerance test is suggestive of insulin resistance in the HFD mice (Figure 2H). This is consistent with previous studies demonstrating insulin resistance based on glucose tolerance tests and hyperinsulinemia even as early as three days after an HFD feeding regimen [16]. The hyperglycemia observed in these mice (211.7 ± 13.5 mg/dL) was in the prediabetes range [26].

The metabolic and biochemical characterization show that -four-week HFD consumption results in a partially developed metabolic syndrome phenotype (Table 1). The HFD mice show a statistically significant increase in plasma total cholesterol (TC), HDL-C, and non-HDL cholesterol (non-HDL-C), over the control mice. HDL-C comprises the majority of the plasma cholesterol pool in the C57Bl/6J mice [27] and an increase in HDL-C upon HFD feeding has been reported previously [28]. Importantly, non-HDL-C, a well-established indicator of pro-atherogenic Apo-B-containing lipoproteins, was increased upon HFD feeding (*p* < 0.05). Additionally, the TC/HDL-C ratio, another measure of cardiovascular risk, showed an increasing trend, although it did not reach statistical significance [29]. Although the biochemical analysis revealed increased hepatic triglyceride (TG) contents, the histological staining of liver sections showed an absence of steatotic changes upon HFD feeding (Supplementary Figure S1). Importantly, the hepatic TGs, although elevated, were at least 5- to 10-fold lower than previously reported steatotic levels after a 12–16-week HFD feeding [30]. Interestingly, while plasma lipoprotein lipase (LPL) activity showed a statistically significant decrease, the plasma TG levels were essentially unchanged. Overall, the 4-week high-fat feeding results in a metabolic profile that is transitional compared to 12–16-week feeding; while some elements of metabolic derangements are evident (hyperglycemia, insulin resistance, cholesterol changes), other aspects (TG changes in the blood and liver) have not yet manifested.

### 2.2. Effect of Short-Term HFD on Plasma HDL Proteome Dynamics

A key effect of HFD-induced chronic low-grade inflammation is an alteration in the functions of the protein cargo of the HDL particles [10]. In addition to apolipoproteins, HDL carries several other protein families including those involved in the complement activation, lipid transfer, acute-phase response, immune response, anti-inflammatory pathways, proteinase inhibitors, and others. Proteins associated with HDL are continuously exchanged with other apolipoprotein B (ApoB)-containing lipoprotein particles, synthesized, and removed from circulation through degradation. Therefore, the static measurements of HDL proteins may not reveal alterations in the dynamics of the HDL proteome. To understand early diet-induced changes in these metabolic diseases and their CVD complications, we examined the effect of four weeks of HFD on plasma HDL proteome dynamics.

Different HDL isolation methods have been used to analyze the HDL proteome. The effect of ApoB depletion, a well-established method for HDL isolation, on the HDL proteome composition has been characterized [31]. It was demonstrated that among multiple ApoB-depletion methods, ApoB-precipitation with dextran sulfate/MgCl_2_ does not affect the HDL proteome composition. Recently, we demonstrated that ApoB-depletion is compatible with the immunocapture method that is known to preserve the HDL composition [32]. Thus, in this study, we used shotgun proteomics to monitor the kinetics of HDL proteins in ApoB-depleted plasma.

Protein turnover was quantified using ^2^H_2_O as a tracer and high-resolution mass spectrometry. Briefly, after three weeks of the diet experiment, each mouse was loaded with a bolus of heavy water (30 μL/g body weight, 99.99% isotopic and chemical purity) followed by 8% (*v/v*) heavy water in drinking water for seven days. According to this method, the time course of ^2^H-enrichment in unique peptides allows the estimation of the fraction of a protein pool that is newly synthesized. The high sensitivity of this method allows the assessment of the HDL dynamics in a single mouse by collecting multiple small blood samples (~60 μL) from the same animal at different time points [33]. The major criteria for the kinetic analysis using this method are a sufficiently high intensity of analyzed isotopomers (10^6^–10^8^ ion counts) and the presence of the same tryptic peptide in 5 to 6 time points that restricts the number of quantifiable proteins.

**Figure 3:**
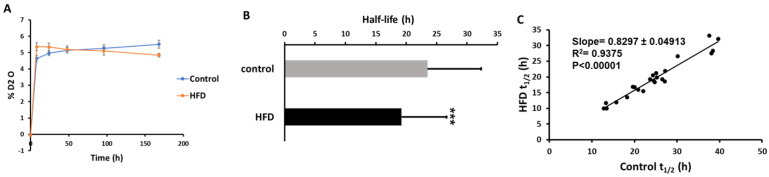
Effect of an HFD on plasma HDL proteome dynamics. **A**, Total body water enrichment. The mice were given a bolus load of heavy water (30 μL/g body weight) and followed by maintenance of 8% (v/v) heavy water in drinking water for seven days which resulted in a steady-state body water enrichment of ~5%. **B**, Comparison of average half-lives of HDL proteins in the control and HFD groups. Results are mean ± SD; n = 6–7/group. Statistical significance, *** indicates *p* < 0.005 compared to control. **C**, The relationships between the half-lives of individual proteins in HFD mice as a function of these values in control mice.

Our labeling protocol resulted in ~5% body water enrichment in mice (Figure 3A). A total of 96 and 89 proteins were identified in ApoB-depleted plasma in all animals from the control and HFD groups, respectively. Overall, 78 of these proteins were present in both groups. Due to the stringent criteria listed above, the kinetics of only 35 proteins could be accurately quantified in all animals from both groups. Each of these proteins has previously been characterized in HDL particles [34]. The proteome dynamics study revealed that the HFD resulted in increased turnover rates (i.e., a reduced half-life) of two-thirds (67%) of all analyzed proteins (Table 2). The average half-life of HDL-associated proteins was significantly reduced in HDL isolated from HFD-fed mice (23.52 ± 8.8 h in control vs. 19.23 ± 7.39 h in HFD, *p* < 0.05) (Figure 3B). This effect of HFD on plasma HDL protein kinetics was also shown in a scatter plot of the half-life of proteins in the HFD group relative to the control group (Figure 3C). In addition to a significant correlation of half-lives of proteins in control and HFD groups (*r*^2^ = 0.938, *p* < 0.0001), the slope of the regression curve (0.83) indicates that the mean half-life of proteins was reduced by ~17% due to four weeks of HFD consumption in mice.

**Figure 4:**
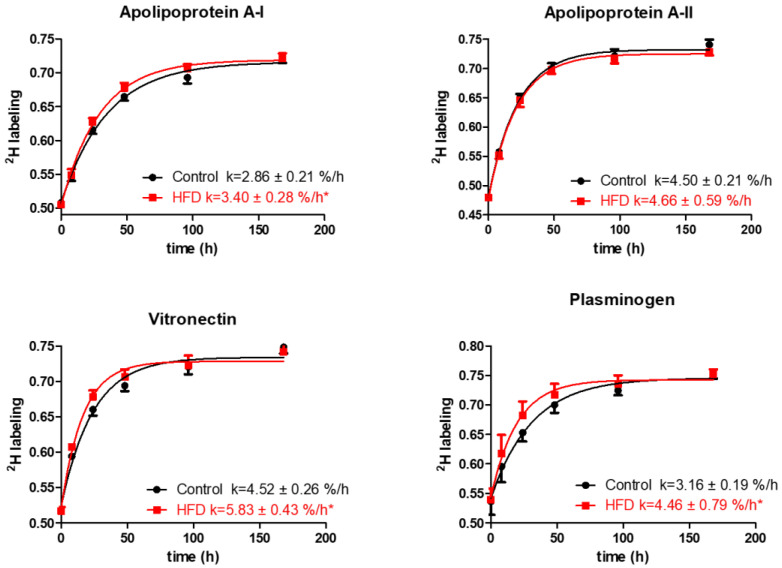
Typical kinetics curves of HDL proteins. The time course of ^2^H-labeling of apolipoprotein A-I, apolipoprotein A-II, vitronectin, and plasminogen peptides is shown for mice fed either the control diet or the HFD groups. The fractional catabolic rate is denoted by the rate constant k. Statistical significance, * indicates *p* < 0.05 compared to control.

The analysis of quantified proteins shows that the HFD resulted in a significant increase in turnover rates of several proteins involved in lipid metabolism and transport, thrombosis, and acute-phase responses. Particularly, the HFD results in an increased turnover of ApoA-I, the principal protein of HDL involved in cholesterol transport (2.86 ± 0.21 vs. 3.40 ± 0.28 %/h, *p* < 0.001, Figure 4). 

Additionally, HFD also increased the turnover rates and reduced half-lives of vitronectin, prothrombin, and plasminogen, the HDL proteins with antithrombotic activities (Figure 4, Table 2). Vitronectin binds to integrin and consequently promotes cell adhesion [35]. It also inhibits the detrimental effects of the complement system on the membranes that result in increased permeability of vessels and the initiation of inflammatory atherosclerotic processes [36]. Plasminogen serves as a precursor for plasmin, a serine protease that helps to dissolve clots in circulation [37]. The increased turnover of these proteins in our study suggests their compensatory enhanced synthesis in response to HFD-induced inflammation. The HFD also significantly increased the turnover rate of albumin, reducing its half-life (76.93 ± 7.49 vs. 66.08 ± 6.13 h, *p* < 0.001) (Table 2). Previous studies have identified albumin as an HDL-associated protein [38,39,40]. However, it also likely that albumin could present as a contaminant in the HDL purification process, although the possibility of such contamination was not investigated in these samples. Similarly, reduced half-lives were observed for ceruloplasmin and serotransferrin and other acute-phase proteins involved in oxidative defense, immune responses, and iron metabolism. Intriguingly, the turnover study also revealed a strong effect of HFD on the kinetics of multiple proteins involved in complement pathway activation. For example, HFD increased the turnover rates of C3 and complement factors B, D, I, and H (*p* < 0.05), components of the alternative complement pathway. Similar boosts in turnover were observed for C8 and clusterin, proteins of the terminal complement pathway (all *p* < 0.05). Although the HFD significantly enhanced the turnover of the majority of the analyzed proteins, the kinetics of some proteins remained unchanged. We did not observe a significant change in the turnover of hemopexin, fibrinogen alpha, and other proteins (Table 2). In addition, HFD had only a modest effect on the turnover of proteins involved in lipid metabolism, including ApoA-II, ApoE, ApoC-I, and ApoC-III.

### 2.3. Pathways Associated with the HFD-Induced Alterations in Plasma HDL Proteome Dynamics

**Figure 5:**
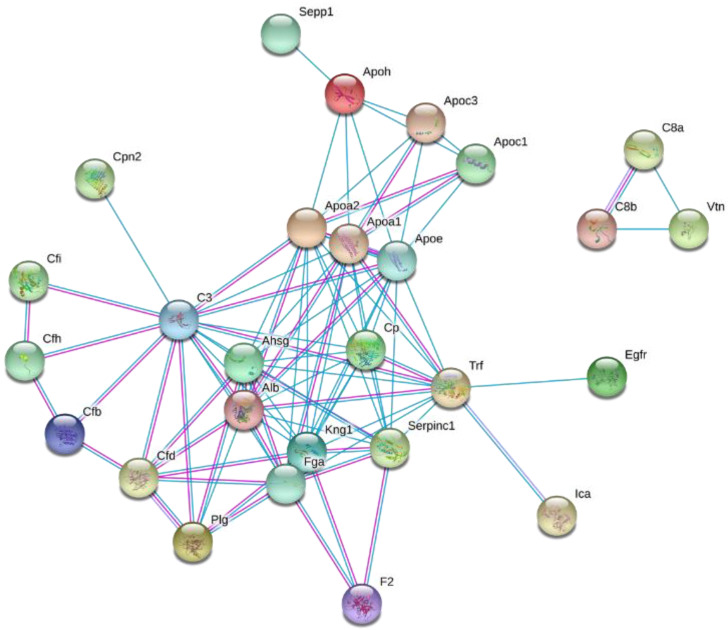
Interaction network of proteins with HFD-induced altered turnover rates. The identified proteins with altered turnover rates were analyzed using the STRING database [41].

To explore the biological significance of the altered plasma HDL proteome dynamics in the context of diet-induced obesity, the changes in protein turnover were further analyzed using STRING [41]. The functional grouping revealed that the majority of the proteins with increased turnover are associated with pathways involved in the innate immunity and inflammation, protein degradation, acute-phase response, and lipid and vitamin transport (all *p* < 0.05) (Figure 5). Consistent with these results, STRING-based pathway analysis suggested HFD-induced activation of the alternative complement pathway (Supplementary Figure S2). Recently, we demonstrated a similar stimulation of the alternative complement pathway in LDLR^-/-^ mice fed a Western-type diet for 12 weeks [42]. The pathway analysis also demonstrated that the HFD significantly increased the flux of proteins involved in the stress response suggesting that the short-term HFD results in a hepatic acute-phase response leading to a pro-inflammatory remodeling of the HDL proteome.

## 3. Discussion

Our findings suggest that significant metabolic disturbances can occur over a relatively short duration of HFD intake in mice. However, the observed absence of hepatic steatosis upon short-term HFD feeding contrasts with the development of NAFLD in the long-term HFD feeding regimens. While insulin resistance is rapidly established upon starting the HFD, a compensatory surge in fatty acid oxidation capacity in the liver is likely able to offset the effects of the enhanced flux of fatty acids to the liver at this early stage of disease progression. In agreement with this notion, we observed a statistically significant increase in the gene expression of lipin-1 and a trend for increased gene expression of carnitine palmitoyltransferase-1 alpha (*Cpt1a*)—both of which stimulate fatty acid oxidation (Supplementary Figure S3). Previous studies have also observed a dichotomous effect of HFD on fatty acid oxidation—an early increase in oxidative capacity followed by a later decrease which eventually leads to the development of NAFLD [43]. In the same study, the expression of *Cpt1a* was shown to mirror the changes in the gene expression of peroxisome proliferator activated receptor alpha (PPARα), which transcriptionally activates *Cpt1a*. While the effect of HFD on PPARα is still debatable [44,45], we speculate that PPARα signaling is part of a compensatory response in the liver to the high dietary fat intake. Overall, there is an apparent lack of hepatic abnormality in mice fed HFD for four weeks, whereas the insulin sensitivity is impaired, dyslipidemia is evident to some extent, and the kinetics of liver-derived proteins are altered, which leads to HDL remodeling. 

This HDL-associated proteome remodeling is characterized by an increased turnover of the acute-phase response proteins and the complement pathway proteins associated with HDL. The observed increase in the turnover rate of these proteins suggests their enhanced synthesis and the activation of these pathways. The association of acute-phase response proteins with HDL has widely been reported [46]. An increased expression of acute-phase response proteins is correlated with pro-inflammatory HDL. A major acute-phase reactant that associates with HDL is serum amyloid alpha-1 (SAA-1). Our proteome dynamics study failed to estimate an HFD-induced change in SAA-1 kinetics due to its low abundance in ApoB-depleted plasma. However, a robust increase in the liver expression of the SAA-1 gene was observed (Supplementary Figure S3). Under the condition of the acute-phase response, SAA-1 proteins assume the role of the primary apolipoprotein of the HDL particle. While healthy HDL protects against LDL oxidation, the acute-phase HDL with increased SAA-1 enhances LDL oxidation in cell culture [47]. Evidence from SAA-1 and SAA-2 knockout mice shows that SAA has a causal role in HDL dysfunction by impairing its anti-inflammatory activity [48].

Additionally, our HDL protein turnover findings reveal a major role of the complement pathway in HFD-induced metabolic dysfunction. Complement C3 levels have been linked with insulin resistance, hepatic steatosis, risk of metabolic syndrome, type 2 diabetes mellitus, and CVD [49,50,51,52]. Overall, the activation of upstream classical and alternative pathways and the common terminal complement pathway are implicated in cardiometabolic diseases [53]. Our findings support the role of complement proteins in metabolic dysregulation including its effect on early HDL remodeling. 

Interestingly, along with the increased flux of HDL proteins, we also noted a significant increase in HDL-C levels upon high-fat feeding. These findings have also been observed in other reported studies performed in rodents as well as in humans [17,28,54,55,56]. The increased concentration of HDL-C following a high-fat feeding protocol is believed to be related to an increase in both the transport rates and the fractional catabolic rate (FCR) for cholesterol-esters and increased translation of ApoA-I [28]. The association between HDL-C and CVD risk is more convoluted than originally proposed, and as such, increased HDL-C levels should be cautiously interpreted. Rather, observations of increased SAA-1 gene expression and the increased flux of acute-phase response proteins suggest a potential pro-inflammatory remodeling of HDL composition and function despite elevated HDL-C levels. Future studies are warranted to assess the functional consequences of altered plasma HDL proteome dynamics. Nevertheless, the observed changes in protein turnover are significant insights into the short-term HFD-induced HDL remodeling process. While lacking absolute protein quantification data can be inferred as a study limitation, it is important to recognize that a protein kinetics analysis is a more sensitive means of detecting disturbances in protein homeostasis as these typically precede alterations in steady-state protein levels.

The evidence for connections between the innate immune response and metabolic dysregulation has grown over the past decade [57]. Under conditions of nutrient excess and obesity, the innate immune system becomes activated and drives the systemic and organ-specific inflammatory response. The central role of inflammasome activation, an innate immunity signaling pathway, in obesity, insulin resistance, type 2 diabetes mellitus, NAFLD, and atherosclerosis has recently become evident [58,59,60]. Interestingly, the SAA proteins are known to activate the inflammasome in macrophages, neurons, and other cell types [61,62]. Similarly, C-reactive protein activates the inflammasome in the endothelial cells [63]. As a critical component of the inflammatory process, the acute-phase response amplifies tissue inflammation, exacerbates tissue damage, and causes metabolic dysregulation that enhances disease complications. Our observations of increased turnover of HDL-associated acute-phase response proteins in mice fed HFD help to support the hypothesis that these proteins contribute to the pathogenesis of the metabolic disease. Whether the activation of the acute-phase response under conditions of high-fat feeding leads to the downstream inflammasome activation in the liver and arteries still remains to be investigated. 

This study was performed in male mice. Several previous studies have demonstrated a greater propensity of male mice to develop an aggravated metabolic phenotype in response to HFD feeding [64,65]. In addition, sex-dependent differences in the responses to proteolytic stress have been reported. [66,67]. Interestingly, females have a lower basal rate of autophagic flux, a major protein degradation pathway [67]. Therefore, male mice may potentially exhibit a higher basal protein turnover rate as compared to females under the conditions of high-fat feeding performed in our study. The effect of gender on HDL proteome dynamics requires further assessment.

In conclusion, the ^2^H_2_O-based metabolic labeling in conjunction with the pathway analysis demonstrates that an early immune response-induced inflammatory remodeling of liver-secreted proteins precedes diet-induced steatosis and dyslipidemia. These findings emphasize the importance of understanding early events in the onset of the full spectrum of metabolic disease induced by HFD feeding. The elucidation of these processes is crucial to early identification and intervention in these widely prevalent diseases.

## 4. Materials and Methods

### 4.1. Animal Experiments

The animal study protocol was approved by the Institutional Animal Care and Use Committee (protocol # 15-010, approval date-10/17/2016) at the Northeast Ohio Medical University (NEOMED). Animal experiments were performed in accordance with the National Institutes of Health (NIH) guidelines. Eight-week-old male C57BL/6J mice (Jackson Laboratories) were adapted to a 12-h light–dark cycle, chow diet (20% kcal from protein, 70% kcal from carbohydrate and 10% kcal from fat, Harlan Teklad), and water. Mice (n = 6–7/group) were fed either a normal chow diet (ND) or an HFD (HFD D12492, 20% kcal from protein, 20% kcal from carbohydrate, and 60% kcal from fat, Research Diets) for 4 weeks. Body weights of mice were measured every two days and food consumption was tracked every day of the feeding period. The fat mass and lean tissue mass were determined in live mice using an EchoMRI 3-in-1 Body Composition Analyzer (EchoMRI; Houston, TX, USA) before the initiation of the feeding period and before sacrificing mice after the feeding period. 

After three weeks of the feeding period, the glucose tolerance test was performed following a 10-h overnight fast. A blood sample was taken prior to (0 min) an intraperitoneal (i.p.) injection with D-Glucose at 2g/kg body weight and subsequent blood samples being collected from the tail vein at 30, 60, 90, and 120 min after glucose injection. Blood glucose was measured using a OneTouch Ultra Mini glucometer (LifeScan; Milpitas, CA, USA). The area under the curve calculation based on the trapezoid rule was performed using the GraphPad prism.

The ^2^H_2_O-based kinetic study was performed during the last week of the diet experiment. To reduce the stress associated with multiple blood sampling, the baseline blood samples were collected through the retro-orbital sinus a week before the kinetic study. Seven days before sacrifice, animals received a bolus dose (i.p. injection) of ^2^H_2_O (30 μL of 99.9% ^2^H-labeled saline per gram body weight), followed by 8% ^2^H_2_O in drinking water. Blood samples (60–80 µL) from the retro-orbital sinus were taken at 8 h, 1 day, 2 days, and 4 days after the initiation of ^2^H_2_O labeling and were collected in potassium ethylenediaminetetraacetic acid (EDTA)-containing tubes (Figure 1). The animals were euthanized seven days after ^2^H_2_O exposure, and the terminal EDTA-preserved blood was collected through the retro-orbital sinus. Plasma was saved at -80 ˚C until the analyses. 

### 4.2. In Vivo LPL Activity

Post-heparin LPL activity was determined in mice at the completion of the feeding period. Mice were injected with heparin (0.5 units/g body weight) 15 min before sacrifice. Blood was collected and LPL activity was determined in the plasma using the Roar-LPL activity assay kit (Roar RB-LPL; Sigma MAK109). 

### 4.3. Lipid Measurements

Terminal fasted samples from fasted mice were collected and lipid content was assayed using cholesterol and triglyceride reagents (Infinity, Thermo Scientific, #TR13421 and #TR22421, respectively). The non-HDL cholesterol level was calculated by subtracting the HDL-C from the TC. Liver tissue (100 mg) was homogenized in methanol and lipids were extracted in a chloroform:methanol (2:1, *v/v*) solvent mixture, as described previously [68]. 

### 4.4. RNA Isolation, cDNA Synthesis, and Quantitative Real-Time Polymerase Chain Reaction (qRT-PCR)

Total RNA from the liver was isolated with TRIzol reagent (ThermoFisher). The reverse transcription reaction (Verso cDNA synthesis kit, AB1453B, ThermoFisher) was performed on 900 ng of the RNA to generate cDNA. Sybr Green (Fermentas) fluorescence was used to perform the PCR reaction. Glyceraldehyde-3-phosphate dehydrogenase (GAPDH) was used as an internal control. Relative mRNA expression was quantified using the ΔΔ*C*_T_ method. The PCR primers used for the amplification of target genes are presented in Supplementary Table S1.

### 4.5. Total Body Water Enrichment Measurements

The ^2^H-enrichment of total body water was measured using a modification of the acetone exchange method as described [69]. Body water enrichment was calculated using the regression equation derived from the analysis of a set of calibration curve samples containing 0 to 5% ^2^H_2_O. 

### 4.6. Isolation of HDL Proteins

HDL was isolated by the depletion of ApoB-containing lipoprotein particles using a commercially available kit (Stanbio Laboratory, Boerne, TX, USA) [33], which allows reproducible tryptic digestion and quantification of plasma proteins associated with HDL [24]. 

### 4.7. Tryptic Digestion of Proteins

Proteins in the supernatant from the ApoB-depleted plasma (25 μL) were used for the proteomics analysis. Proteins were precipitated with 1 mL of cold acetone (−20 °C); the protein pellets were digested with trypsin as described [70]. Protein digests were isolated using a solid-phase extraction on the Pierce Pepclean C18 spin columns. Samples were reconstituted in 0.1% formic acid and 2% of acetonitrile (ACN) and analyzed by nanospray LC-MS/MS. 

### 4.8. Tandem Mass Spectrometry Analysis

A solution containing the tryptic peptides was fractionated using Ultimate 3000 UHPLC (Thermo Scientific, Waltham, MA, USA) and analyzed on a Q Exactive™ Plus Hybrid Quadrupole-Orbitrap™ Mass Spectrometer (Thermo Scientific, CA, USA). The samples were desalted on an Acclaim PepMap100 precolumn (300 μm × 5 mm, C18, 5 μm, 100Å, Thermo Fisher Scientific), and then peptides were introduced to an Acclaim PepMap RSLC reverse-phase nanocolumn (75 μm × 15 cm, C18, 2 μm, 100Å, Thermo Fisher Scientific) at 300 nL/min with a mobile phase A (0.1% formic acid and 2% ACN in water) and B (20% water in ACN with 0.1% formic acid). For the chromatographic separation of tryptic peptides, a 120-min gradient was employed with 2% of mobile phase B. After 4 min of desalting, mobile phase B was linearly increased to 40% in 100 min. Mobile phase B was then ramped to 90% in 5 min and then held at 90% B for 10 min. Subsequently, mobile phase B was decreased to 2% for 2 min and equilibrated for 13 min with 2% of phase B.

A tandem mass spectrometry analysis was performed with a 70,000 resolution (200 m/z) and m/z 380–1300 (MS) range. The fragment ions were generated by higher-energy collisional dissociation (HCD) performed at a normalized collision energy of 27%. MS/MS spectra were collected in the data-dependent analysis mode for the 15 most abundant product ions with an isolation window of 1.4 m/z and offset of 0.3 m/z at a 17,500 resolution (200 m/z). The automatic gain control (AGC) target was set at 3.0 × 10^6^ and 2.0 × 10^4^ ions for MS and MS/MS scans, respectively. Dynamic exclusion was enabled for a duration of 20 s.

### 4.9. Proteome Dynamics Analysis

For the characterization of HDL proteins, all the spectra obtained from the mass spectrometer were transformed into an mzML file format. The data were searched using Mascot software (Matrix Science, London, UK) version 2.3 against the mouse subset of the SwissProt protein database released in October 2016 (containing 552,884 entries) with cysteine carbamidomethylation as a fixed modification and methionine oxidation as a variable modification and trypsin digestion with a maximum of two missed cleavages per peptide. ^2^H-labeling of the peptides precursor ions from high-resolution full scan (MS1) spectra was extracted for each isotopomer [22]. Isotopomers are molecules that differ by the presence of isotopes in different positions resulting in an isotopomer pattern in a mass spectrum. The ^2^H-enrichment of analyzed peptides was calculated as described below.

### 4.10. Turnover Rate Constant and Half-Life Calculation

The protein synthesis was calculated based on the ^2^H-enrichment of tryptic peptides [71]. The rate of the ^2^H-labeling of unique proteolytic peptides of a protein represents the synthesis rate of that protein. The total ^2^H-labeling of peptides (I(t)) is expressed as shown below in Equation (1) [71]:

(1) $$I\left(t\right)=1-Io\left(t\right)=\;\overset{n}{\underset{j=1}{\sum }}M_{j}\left(t\right)/\overset{n}{\underset{j=0}{\sum }}M_{j}\left(t\right)$$

The rate constant (*k*) was determined using single compartmental kinetic modeling. A time course of ^2^H-labeling (*I*(*t*)) of peptides was fitted to an exponential rise curve equation after the removal of statistical outliers using the Prism software [71]:

*I*(*t*) = *I*(0) + (*I*(*plateau*) − *I*(0)) × (1 − *e*^−*kt*^) (2)

In this equation, *I*(0) and *I*(*plateau*) represent the total labeling at the baseline (*t* = 0) and plateau, respectively.

Our calculations assume the metabolic steady-state when the total content of a protein (pool size) does not change. In this condition, the fractional synthesis rate (FSR) is equal to FCR and both are expressed as the rate constant. The data from multiple unique peptides were averaged to calculate the mean rate constant and half-life (*t½* = *ln*2/*k*) of a protein.

### 4.11. Network Analysis

The kinetic data from the proteome dynamics experiments were further analyzed by STRING (string-db.org) [41]. Protein–protein interaction networks identified the proteins with altered turnover rates from the STRING library of proteins. This analysis allows the identification of networks of proteins with related biological functions.

### 4.12. Data Accession

The mass spectrometry proteomics data have been deposited to the ProteomeXchange Consortium via the PRIDE [72] partner repository with the dataset identifier PXD010272.

### 4.13. Statistical Analysis

To evaluate the effect of a short-term HFD on plasma HDL proteome dynamics, we compared the rate constant values calculated for different proteins in each mouse (n = 6–7/group) determined from the time course of ^2^H-labeling of several (n = 3–20) peptides unique to each protein. For this purpose, all six time points in each mouse (n = 6–7/group) were fitted into the first-order kinetic equation (Equation (2)). The results from the unique peptides were averaged to calculate the final turnover rate of protein in each mouse. A two-tailed Student *t*-test or two-way ANOVA with repeated measures (for the GTT) were used for the determination of statistical significance, with *p* < 0.05 considered as statistically significant.

## Figures and Tables

**Table 1:** Biochemical characteristics of control and HFD-fed mice. C57BL/6J male mice were fed a control diet or a high-fat diet for four weeks. After an 8-h fast, the mice were sacrificed, and blood and tissue samples were collected for biochemical analysis.

	Control Diet	High-Fat Diet	*p*-Value
Body weight (g)	25.4 ± 0.4	28.2 ± 0.7	<0.005
Liver weight (g)	1.04 ± 0.03	0.85 ± 0.02	<0.005
Liver weight/Body weight (%)	4.27 ± 0.10	3.01 ± 0.06	<0.005
Blood glucose (mg/dL)	106.5 ± 7.5	211.7 ± 13.5	<0005
Plasma TG (mg/dL)	35.5 ± 2.1	36.7 ± 2.4	0.718
Plasma LPL activity (nmol FFA/min/mL)	362.6 ± 11.5	312.6 ± 7.7	<0.005
Plasma TC (mg/dL)	87.3 ± 2.7	139.9 ± 6.3	<0.005
Plasma HDL-C (mg/dL)	69.9 ± 3.0	97.3 ± 6.5	<0.005
Plasma non-HDL-C (mg/dL) (calculated)	17.5 ± 4.2	49.3 ± 10.9	<0.05
TC/HDL-C ratio	1.26 ± 0.06	1.54 ± 0.14	0.120
Hepatic TG (μg/mg liver tissue)	11.3 ± 0.4	14.3 ± 0.9	<0.05

HDL-C: high-density lipoprotein-cholesterol; LPL: lipoprotein lipase; TC: total cholesterol; TG: triglycerides.

**Table 2:** Half-lives of HDL proteins.

Accession (Uniprot)	Protein	Molecular Weight (kDa)	Function ^#^	Control	HFD
				Mean t½ (hours ±SD)	Mean t½ (hours ±SD)
Q546G4	Albumin	68.7	Chaperone binding; DNA binding; drug binding; enzyme binding; fatty acid binding; identical protein binding	76.93 ± 7.49	66.09 ± 6.13 *
P29699	Alpha-2-HS-glycoprotein	37.3	Cysteine-type endopeptidase inhibitor activity; endopeptidase inhibitor activity; receptor signaling protein tyrosine kinase inhibitor activity	20.92 ± 1.60	15.92 ± 2.74 *
A0A0A6YWH7	Antithrombin-III (fragment)	52.0	Heparin binding; peptidase inhibitor activity; protease binding; serine-type endopeptidase inhibitor activity	24.77 ± 2.13	18.34 ± 1.16 *
Q3V2G1	Apolipoprotein A-I	30.6	Amyloid-beta binding; apolipoprotein A-I receptor binding; apolipoprotein receptor binding; chemorepellent activity; cholesterol binding	24.33 ± 1.76	20.51 ± 1.64 *
Q6LD55	Apolipoprotein A-II	11.3	Apolipoprotein receptor binding; cholesterol binding; cholesterol transporter activity; heat shock protein binding; high-density lipoprotein particle binding	15.43 ± 0.69	15.09 ± 1.68
Q6LAL7	Beta-2-glycoprotein 1	38.6	Heparin binding; identical protein binding; lipid binding; phospholipid binding	30.88 ± 3.46	20.75 ± 4.67 *
Q9DBB9	Carboxypeptidase N subunit 2	60.5	Enzyme regulator activity; metallocarboxypeptidase activity	30.14 ± 2.26	26.60 ± 2.00 *
P01027	Complement C3	186.5	C5L2 anaphylatoxin chemotactic receptor binding; cofactor binding; endopeptidase inhibitor activity; lipid binding; protein binding	12.77 ± 0.81	9.98 ± 1.20 *
Q3UEG8	Complement Factor B	85.5	Complement binding; protein binding; serine-type endopeptidase activity	12.71 ± 1.01	10.85 ± 1.04
D6RGQ0	Complement Factor H	139.1	Complement component C3b binding; heparan sulfate proteoglycan binding; heparin binding; protein binding	19.60 ± 1.84	16.80 ± 1.58 *
Q61129	Complement Factor I	67.3	Hydrolase activity; metal ion binding; peptidase activity; scavenger receptor activity; serine-type endopeptidase activity	25.25 ± 1.29	19.88 ± 1.50 *
Q91X72	Hemopexin	51.3	Cellular iron ion homeostasis; heme metabolic process; heme transport; hemoglobin metabolic process; positive regulation of humoral immune response mediated by circulating immunoglobulin	39.82 ± 6.57	39.92 ± 9.65
A0A0R4J039	Histidine-rich Glycoprotein	59.6	Cysteine-type endopeptidase inhibitor activity; heme binding; heparan sulfate proteoglycan binding; heparin binding; immunoglobulin binding	23.63 ± 1.99	19.28 ± 2.83 *
A0A0R4J038	Kininogen-1	73.1	Cysteine-type endopeptidase inhibitor activity; peptidase inhibitor activity; receptor binding	25.09 ± 1.59	21.18 ± 0.91 *
P28665	Murinoglobulin-1	165.3	Endopeptidase inhibitor activity; peptidase inhibitor activity; serine-type endopeptidase inhibitor activity	39.67 ± 3.22	32.08 ± 1.73 *
Q3V1T9	Plasminogen	90.8	Apolipoprotein binding; chaperone binding; endopeptidase activity; enzyme binding; hydrolase activity	22.04 ± 1.25	15.45 ± 3.42 *
Q61838	Pregnancy Zone Protein	165.8	Brain-derived neurotrophic factor binding; endopeptidase inhibitor activity; nerve growth factor binding; peptidase inhibitor activity; protein complex binding	41.71 ± 3.50	38.91 ± 2.29
Q07456	Protein AMBP	39.0	Heme binding; IgA binding; peptidase inhibitor activity; protein homodimerization activity; serine-type endopeptidase inhibitor activity	14.23 ± 1.97	13.21 ± 1.09
Q3TJ94	Prothrombin	70.3	Calcium ion binding; heparin binding; hydrolase activity; lipopolysaccharide binding; peptidase activity	27.20 ± 6.04	21.87 ± 4.66 *
Q921I1	Serotransferrin	77.0	Ferric iron binding; ferric iron transmembrane transporter activity; ferrous iron binding; protein binding	37.62 ± 1.74	33.12 ± 6.63 *
P21614	Vitamin D-binding Protein	53.6	Actin binding; vitamin D binding; vitamin transporter activity	13.48 ± 0.50	10.00 ± 0.84 *
G3X8Q5	Ceruloplasmin	121.1	Chaperone binding; copper ion binding; ferroxidase activity; metal ion binding; oxidoreductase activity	27.10 ± 6.12	18.55 ± 3.41 *
P34928	Apolipoprotein C-I	9.3	Fatty acid binding; lipase inhibitor activity; phosphatidylcholine binding; phospholipase inhibitor activity	17.66 ± 6.52	10.39 ± 8.06
P33622	Apolipoprotein C-III	10.9	Lipase inhibitor activity; lipid binding; phospholipid binding	10.16 ± 1.57	11.30 ± 2.18
P08226	Apolipoprotein E	35.9	Amyloid-beta binding; antioxidant activity; cholesterol binding; cholesterol transporter activity; heparin binding	10.50 ± 5.53	8.09 ± 1.95
A2A997	Complement component C8 Alpha Chain	66.1	Complement binding; protein complex binding	26.50 ± 2.22	19.24 ± 3.85 *
Q9QWK4	CD5 Antigen-Like	38.9	Scavenger receptor activity	32.66 ± 8.35	28.42 ± 3.43
Q8BH35	Complement component C8 beta chain	66.2	Constituent of the membrane attack complex (MAC) that plays a key role in the innate and adaptive immune response by forming pores in the plasma membrane of target cells	24.51 ± 4.68	18.83 ± 1.95 *
Q9EP98	Epidermal growth factor receptor	138.4	Protein tyrosine kinase activity	38.05 ± 3.82	27.58 ± 3.90 *
P03953	Complement Factor D	28.1	actor D cleaves factor B when the latter is complexed with factor C3b, activating the C3bbb complex, which then becomes the C3 convertase of the alternate pathway. Its function is homologous to that of C1s in the classical pathway	20.06 ± 1.19	16.71 ± 0.61 *
A0A075B5P6	Ig mu chain C region (fragments)	50.1	Immunoglobin receptor binding	44.95 ± 8.23	35.34 ± 11.15
Q9DBD0	Inhibitor of Carbonic Anhydrase	76.7	Inhibitor for carbonic anhydrase 2 (CA2). Does not bind iron ions	18.24 ± 1.36	13.52 ± 0.41 *
P29788	Vitronectin	54.9	Interact with glycosaminoglycans and proteoglycans. Is recognized by certain members of the integrin family and serves as a cell-to-substrate adhesion molecule. Inhibitor of the membrane-damaging effect of the terminal cytolytic complement pathway.	15.71 ± 2.27	11.95 ± 0.88 *
E9PV24	Fibrinogen alpha chain	87.4		40.15 ± 7.35	41.78 ± 3.03

HFD: High-Fat Diet; HDL: high-density lipoprotein. ^#^ These functions were curated from the UNIPROT database. * *p* < 0.05 vs. control.

## Supplementary Materials

Supplementary materials can be found at https://www.mdpi.com/1422-0067/21/20/7472/s1. Figure S1: Representative images of the isolated liver tissue and hematoxylin and eosin (H&E) stained liver sections from mice fed either a control diet or a high-fat diet for 4-weeks. The staining shows a lack of steatotic changes in the liver after four weeks of high-fat feeding. Figure S2: Representation of the protein-protein interaction networks of complement activation. Functional enrichment analysis of the proteins identified in the HDL proteome dynamics study revealed increased turnover rates of alternative and terminal complement activation pathways (denoted in red) upon high-fat feeding in wild type mice for four weeks. Figure S3: Gene expression analysis using RNA isolated from livers of mice fed either the control diet or the high-fat diet. At the end of the study, the livers from the mice were harvested and RNA was isolated. Complementary DNA (cDNA) was synthesized from the RNA and the quantitative real-time polymerase chain reaction (qRT-PCR) was performed. The relative expression of indicated genes was calculated using the Ct method. Statistical significance, ** indicates *p* < 0.01, n = 6–7/group. Table S1: Primer sequences used for quantitative real-time polymerase chain reaction (qRT-PCR).

## References

[1] Atawia R.T., Bunch K.L., Toque H.A., Caldwell R.B., Caldwell R.W. (2019). Mechanisms of obesity-induced metabolic and vascular dysfunctions. Front. Biosci..

[2] Herrington W., Lacey B., Sherliker P., Armitage J., Lewington S. (2016). Epidemiology of atherosclerosis and the potential to reduce the global burden of atherothrombotic disease. Circ. Res..

[3] Upadhyay R.K. (2015). Emerging risk biomarkers in cardiovascular diseases and disorders. J. Lipids.

[4] Thomas M.R., Lip G.Y. (2017). Novel risk markers and risk assessments for cardiovascular disease. Circ. Res..

[5] Wang C.Y., Liao J.K. (2012). A mouse model of diet-induced obesity and insulin resistance. Methods Mol. Biol..

[6] Hotamisligil G.S. (2006). Inflammation and metabolic disorders. Nature.

[7] Libby P. (2012). Inflammation in atherosclerosis. Arterioscler. Thromb. Vasc. Biol..

[8] Ronsein G.E., Vaisar T. (2019). Deepening our understanding of HDL proteome. Expert Rev. Proteom..

[9] Ronsein G.E., Vaisar T. (2017). Inflammation, remodeling, and other factors affecting HDL cholesterol efflux. Curr. Opin. Lipidol..

[10] Feingold K.R., Grunfeld C. (2016). Effect of inflammation on HDL structure and function. Curr. Opin. Lipidol..

[11] Jovicic N., Jeftic I., Jovanovic I., Radosavljevic G., Arsenijevic N., Lukic M.L., Pejnovic N. (2015). Differential immunometabolic phenotype in Th1 and Th2 dominant mouse strains in response to high-fat feeding. PLoS ONE.

[12] Hariri N., Thibault L. (2010). High-fat diet-induced obesity in animal models. Nutr. Res. Rev..

[13] Rossmeisl M., Rim J.S., Koza R.A., Kozak L.P. (2003). Variation in type 2 diabetes-related traits in mouse strains susceptible to diet-induced obesity. Diabetes.

[14] Holmes A., Coppey L.J., Davidson E.P., Yorek M.A. (2015). Rat models of diet-induced obesity and high fat/low dose streptozotocin type 2 diabetes: Effect of reversal of high fat diet compared to treatment with enalapril or menhaden oil on glucose utilization and neuropathic endpoints. J. Diabetes Res..

[15] Lutz T.A., Woods S.C. (2012). Overview of animal models of obesity. Curr. Protoc. Pharmacol..

[16] Lee Y.S., Li P., Huh J.Y., Hwang I.J., Lu M., Kim J.I., Ham M., Talukdar S., Chen A., Lu W.J. (2011). Inflammation is necessary for long-term but not short-term high-fat diet-induced insulin resistance. Diabetes.

[17] Williams L.M., Campbell F.M., Drew J.E., Koch C., Hoggard N., Rees W.D., Kamolrat T., Thi Ngo H., Steffensen I.L., Gray S.R. (2014). The development of diet-induced obesity and glucose intolerance in c57bl/6 mice on a high-fat diet consists of distinct phases. PLoS ONE.

[18] Lai Y.S., Chen W.C., Kuo T.C., Ho C.T., Kuo C.H., Tseng Y.J., Lu K.H., Lin S.H., Panyod S., Sheen L.Y. (2015). Mass-spectrometry-based serum metabolomics of a c57bl/6j mouse model of high-fat-diet-induced non-alcoholic fatty liver disease development. J. Agric. Food Chem..

[19] Kashyap S.R., Osme A., Ilchenko S., Golizeh M., Lee K., Wang S., Bena J., Previs S.F., Smith J.D., Kasumov T. (2018). Glycation reduces the stability of apoai and increases HDL dysfunction in diet-controlled type 2 diabetes. J. Clin. Endocrinol. Metab..

[20] Rosenson R.S., Brewer H.B., Ansell B.J., Barter P., Chapman M.J., Heinecke J.W., Kontush A., Tall A.R., Webb N.R. (2016). Dysfunctional HDL and atherosclerotic cardiovascular disease. Nat. Rev. Cardiol..

[21] McCullough A., Previs S.F., Dasarathy J., Lee K., Osme A., Kim C., Ilchenko S., Lorkowski S.W., Smith J.D., Dasarathy S. (2019). HDL flux is higher in patients with nonalcoholic fatty liver disease. Am. J. Physiol. Endocrinol. Metab..

[22] Sadygov R.G., Avva J., Rahman M., Lee K., Ilchenko S., Kasumov T., Borzou A. (2018). D2ome, software for in vivo protein turnover analysis using heavy water labeling and lc-ms, reveals remodeling of hepatic proteome dynamics in a mouse model of NAFLD. J. Proteome Res..

[23] Kasumov T., Li L., Li M., Gulshan K., Kirwan J.P., Liu X., Previs S., Willard B., Smith J.D., McCullough A. (2015). Ceramide as a mediator of non-alcoholic fatty liver disease and associated atherosclerosis. PLoS ONE.

[24] Li L., Bebek G., Previs S.F., Smith J.D., Sadygov R.G., McCullough A.J., Willard B., Kasumov T. (2016). Proteome dynamics reveals pro-inflammatory remodeling of plasma proteome in a mouse model of NAFLD. J. Proteome Res..

[25] Lee K., Haddad A., Osme A., Kim C., Borzou A., Ilchenko S., Allende D., Dasarathy S., McCullough A., Sadygov R.G. (2018). Hepatic mitochondrial defects in a nonalcoholic fatty liver disease mouse model are associated with increased degradation of oxidative phosphorylation subunits. Mol. Cell Proteom..

[26] Fajardo R.J., Karim L., Calley V.I., Bouxsein M.L. (2014). A review of rodent models of type 2 diabetic skeletal fragility. J. Bone Miner. Res..

[27] Yin W., Carballo-Jane E., McLaren D.G., Mendoza V.H., Gagen K., Geoghagen N.S., McNamara L.A., Gorski J.N., Eiermann G.J., Petrov A. (2012). Plasma lipid profiling across species for the identification of optimal animal models of human dyslipidemia. J. Lipid Res..

[28] Hayek T., Ito Y., Azrolan N., Verdery R.B., Aalto-Setala K., Walsh A., Breslow J.L. (1993). Dietary fat increases high density lipoprotein (HDL) levels both by increasing the transport rates and decreasing the fractional catabolic rates of HDL cholesterol ester and apolipoprotein (apo) a-i. Presentation of a new animal model and mechanistic studies in human apo a-i transgenic and control mice. J. Clin. Investig..

[29] Brunner F.J., Waldeyer C., Ojeda F., Salomaa V., Kee F., Sans S., Thorand B., Giampaoli S., Brambilla P., Tunstall-Pedoe H. (2019). Application of non-HDL cholesterol for population-based cardiovascular risk stratification: Results from the multinational cardiovascular risk consortium. Lancet.

[30] Pan X., Zhang Y., Kim H.G., Liangpunsakul S., Dong X.C. (2017). FOXO transcription factors protect against the diet-induced fatty liver disease. Sci. Rep..

[31] Davidson W.S., Heink A., Sexmith H., Melchior J.T., Gordon S.M., Kuklenyik Z., Woollett L., Barr J.R., Jones J.I., Toth C.A. (2016). The effects of apolipoprotein B depletion on HDL subspecies composition and function. J. Lipid Res..

[32] Kheniser K.G., Osme A., Kim C., Ilchenko S., Kasumov T., Kashyap S.R. (2020). Temporal dynamics of high-density lipoprotein proteome in diet-controlled subjects with type 2 diabetes. Biomolecules.

[33] Kasumov T., Willard B., Li L., Li M., Conger H., Buffa J.A., Previs S., McCullough A., Hazen S.L., Smith J.D. (2013). ^2^H_2_O-based high-density lipoprotein turnover method for the assessment of dynamic high-density lipoprotein function in mice. Arterioscler. Thromb. Vasc. Biol..

[34] Shah A.S., Tan L., Long J.L., Davidson W.S. (2013). Proteomic diversity of high density lipoproteins: Our emerging understanding of its importance in lipid transport and beyond. J. Lipid Res..

[35] Preissner K.T., Reuning U. (2011). Vitronectin in vascular context: Facets of a multitalented matricellular protein. Semin. Thromb. Hemost..

[36] Ekmekci O.B., Ekmekci H. (2006). Vitronectin in atherosclerotic disease. Clin. Chim. Acta.

[37] Heissig B., Salama Y., Takahashi S., Osada T., Hattori K. (2020). The multifaceted role of plasminogen in inflammation. Cell Signal..

[38] Heller M., Stalder D., Schlappritzi E., Hayn G., Matter U., Haeberli A. (2005). Mass spectrometry-based analytical tools for the molecular protein characterization of human plasma lipoproteins. Proteomics.

[39] Vaisar T., Pennathur S., Green P.S., Gharib S.A., Hoofnagle A.N., Cheung M.C., Byun J., Vuletic S., Kassim S., Singh P. (2007). Shotgun proteomics implicates protease inhibition and complement activation in the antiinflammatory properties of HDL. J. Clin. Investig..

[40] Davidson W.S., Silva R.A., Chantepie S., Lagor W.R., Chapman M.J., Kontush A. (2009). Proteomic analysis of defined HDL subpopulations reveals particle-specific protein clusters: Relevance to antioxidative function. Arterioscler. Thromb. Vasc. Biol..

[41] Szklarczyk D., Morris J.H., Cook H., Kuhn M., Wyder S., Simonovic M., Santos A., Doncheva N.T., Roth A., Bork P. (2017). The string database in 2017: Quality-controlled protein-protein association networks, made broadly accessible. Nucleic Acids Res..

[42] Li L., Willard B., Rachdaoui N., Kirwan J.P., Sadygov R.G., Stanley W.C., Previs S., McCullough A.J., Kasumov T. (2012). Plasma proteome dynamics: Analysis of lipoproteins and acute phase response proteins with 2H2O metabolic labeling. Mol. Cell Proteom..

[43] Chan M.Y., Zhao Y., Heng C.K. (2008). Sequential responses to high-fat and high-calorie feeding in an obese mouse model. Obesity (Silver Spring).

[44] Lee Y., Wang M.Y., Kakuma T., Wang Z.W., Babcock E., McCorkle K., Higa M., Zhou Y.T., Unger R.H. (2001). Liporegulation in diet-induced obesity. The antisteatotic role of hyperleptinemia. J. Biol. Chem..

[45] Sun B., Jia Y., Hong J., Sun Q., Gao S., Hu Y., Zhao N., Zhao R. (2018). Sodium butyrate ameliorates high-fat-diet-induced non-alcoholic fatty liver disease through peroxisome proliferator-activated receptor alpha-mediated activation of beta oxidation and suppression of inflammation. J. Agric. Food Chem..

[46] Jahangiri A., de Beer M.C., Noffsinger V., Tannock L.R., Ramaiah C., Webb N.R., van der Westhuyzen D.R., de Beer F.C. (2009). Hdl remodeling during the acute phase response. Arterioscler. Thromb. Vasc. Biol..

[47] Navab M., Hama-Levy S., Van Lenten B.J., Fonarow G.C., Cardinez C.J., Castellani L.W., Brennan M.L., Lusis A.J., Fogelman A.M., La Du B.N. (1997). Mildly oxidized ldl induces an increased apolipoprotein j/paraoxonase ratio. J. Clin. Investig..

[48] Han C.Y., Tang C., Guevara M.E., Wei H., Wietecha T., Shao B., Subramanian S., Omer M., Wang S., O’Brien K.D. (2016). Serum amyloid a impairs the antiinflammatory properties of HDL. J. Clin. Investig..

[49] Barbu A., Hamad O.A., Lind L., Ekdahl K.N., Nilsson B. (2015). The role of complement factor c3 in lipid metabolism. Mol. Immunol..

[50] Engstrom G., Hedblad B., Eriksson K.F., Janzon L., Lindgarde F. (2005). Complement. c3 is a risk factor for the development of diabetes: A population-based cohort study. Diabetes.

[51] Kolev M., Kemper C. (2017). Keeping it all going-complement meets metabolism. Front. Immunol..

[52] Onat A., Hergenc G., Can G., Kaya Z., Yuksel H. (2010). Serum complement c3: A determinant of cardiometabolic risk, additive to the metabolic syndrome, in middle-aged population. Metabolism.

[53] Hertle E., Stehouwer C.D., van Greevenbroek M.M. (2014). The complement system in human cardiometabolic disease. Mol. Immunol..

[54] Arisqueta L., Navarro-Imaz H., Labiano I., Rueda Y., Fresnedo O. (2018). High.-fat diet overfeeding promotes nondetrimental liver steatosis in female mice. Am. J. Physiol. Gastrointest. Liver Physiol..

[55] Wolf G. (1996). High-fat, high-cholesterol diet raises plasma HDL cholesterol: Studies on the mechanism of this effect. Nutr. Rev..

[56] Siri-Tarino P.W. (2011). Effects of diet on high-density lipoprotein cholesterol. Curr. Atheroscler. Rep..

[57] Lackey D.E., Olefsky J.M. (2016). Regulation of metabolism by the innate immune system. Nat. Rev. Endocrinol..

[58] Stienstra R., van Diepen J.A., Tack C.J., Zaki M.H., van de Veerdonk F.L., Perera D., Neale G.A., Hooiveld G.J., Hijmans A., Vroegrijk I. (2011). Inflammasome is a central player in the induction of obesity and insulin resistance. Proc. Natl. Acad. Sci. USA.

[59] Grant R.W., Dixit V.D. (2013). Mechanisms of disease: Inflammasome activation and the development of type 2 diabetes. Front. Immunol..

[60] Jin Y., Fu J. (2019). Novel insights into the nlrp 3 inflammasome in atherosclerosis. J. Am. Heart Assoc..

[61] Shridas P., De Beer M.C., Webb N.R. (2018). High-density lipoprotein inhibits serum amyloid a-mediated reactive oxygen species generation and nlrp3 inflammasome activation. J. Biol. Chem..

[62] Yu J., Zhu H., Taheri S., Mondy W., Bonilha L., Magwood G.S., Lackland D., Adams R.J., Kindy M.S. (2019). Serum amyloid a-mediated inflammasome activation of microglial cells in cerebral ischemia. J. Neurosci..

[63] Bian F., Yang X.Y., Xu G., Zheng T., Jin S. (2019). CRP-induced NLRP3 inflammasome activation increases ldl transcytosis across endothelial cells. Front. Pharmacol..

[64] Hwang L.L., Wang C.H., Li T.L., Chang S.D., Lin L.C., Chen C.P., Chen C.T., Liang K.C., Ho I.K., Yang W.S. (2010). Sex differences in high-fat diet-induced obesity, metabolic alterations and learning, and synaptic plasticity deficits in mice. Obesity (Silver Spring).

[65] Pettersson U.S., Walden T.B., Carlsson P.O., Jansson L., Phillipson M. (2012). Female mice are protected against high-fat diet induced metabolic syndrome and increase the regulatory t cell population in adipose tissue. PLoS ONE.

[66] Tower J., Pomatto L.C.D., Davies K.J.A. (2020). Sex differences in the response to oxidative and proteolytic stress. Redox Biol..

[67] Congdon E.E. (2018). Sex differences in autophagy contribute to female vulnerability in alzheimer’s disease. Front. Neurosci..

[68] Bligh E.G., Dyer W.J. (1959). A rapid method of total lipid extraction and purification. Can. J. Biochem. Physiol..

[69] Mahsut A., Wang S.P., McLaren D.G., Bhat G., Herath K., Miller P.L., Hubbard B.K., Johns D.G., Previs S.F., Roddy T.P. (2011). Headspace analyses of ^2^H labeling of acetone: Enabling studies of fatty acid oxidation in vivo. Anal. Biochem..

[70] Walsh M.T., Atkinson D. (1983). Solubilization of low-density lipoprotein with sodium deoxycholate and recombination of apoprotein b with dimyristoylphosphatidylcholine. Biochemistry.

[71] Kasumov T., Ilchenko S., Li L., Rachdaoui N., Sadygov R.G., Willard B., McCullough A.J., Previs S. (2011). Measuring protein synthesis using metabolic ^2^H labeling, high-resolution mass spectrometry, and an algorithm. Anal. Biochem..

[72] Vizcaino J.A., Csordas A., Del-Toro N., Dianes J.A., Griss J., Lavidas I., Mayer G., Perez-Riverol Y., Reisinger F., Ternent T. (2016). 2016 update of the pride database and its related tools. Nucleic Acids Res..

