# Supplementary material for: Early Pro-Inflammatory Remodeling of HDL Proteome in a Model of Diet-Induced Obesity: 2H2O-Metabolic Labeling-Based Kinetic Approach
Source: Int J Mol Sci. 2020 Oct 10;21(20):7472. doi: 10.3390/ijms21207472 (PMC7656294; doi:10.3390/ijms21207472)
Supplement: Supplementary file 1 [file ijms-21-07472-s001.pdf]

Early pro-inflammatory remodeling of HDL proteome in a model of diet-induced obesity: $^{2}H_2O$-metabolic labeling based kinetic approach

Prabodh Sadana*[1], Li Lin[2], Mirjavid Aghayev[2], Serguei Ilchenko[2], Takhar Kasumov*[2]

[1]Department of Pharmacy Practice, College of Pharmacy, Northeast Ohio Medical University, Rootstown, OH

[2]Department of Pharmaceutical Sciences, College of Pharmacy, Northeast Ohio Medical University, Rootstown, OH

*Corresponding authors: Prabodh Sadana (psadana@neomed.edu), Takhar Kasumov (tkasumov@neomed.edu)

Supplementary data

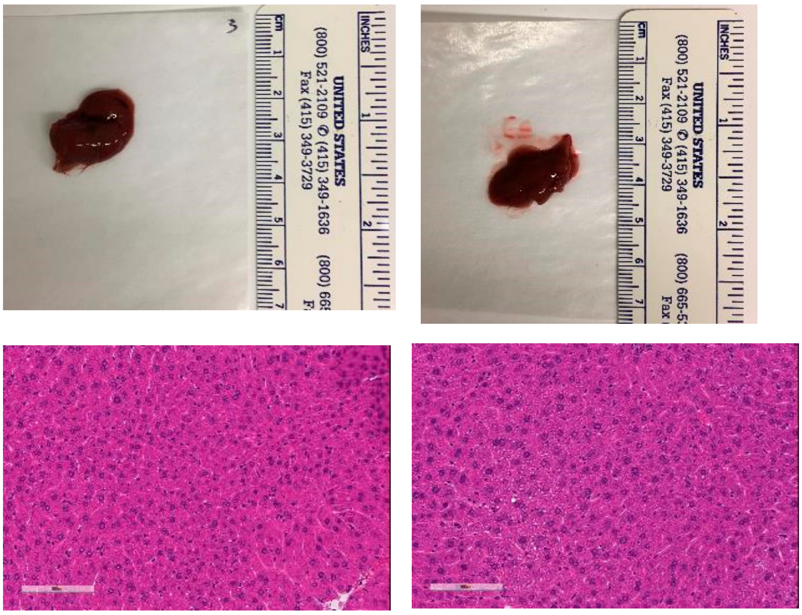

Control HFD

Supplementary Figure S1: Representative images of the isolated liver tissue and hematoxylin and eosin (H&E) stained liver sections from mice fed either a control diet or a high-fat diet for 4-weeks. The staining shows a lack of steatotic changes in the liver after four weeks of high-fat feeding.

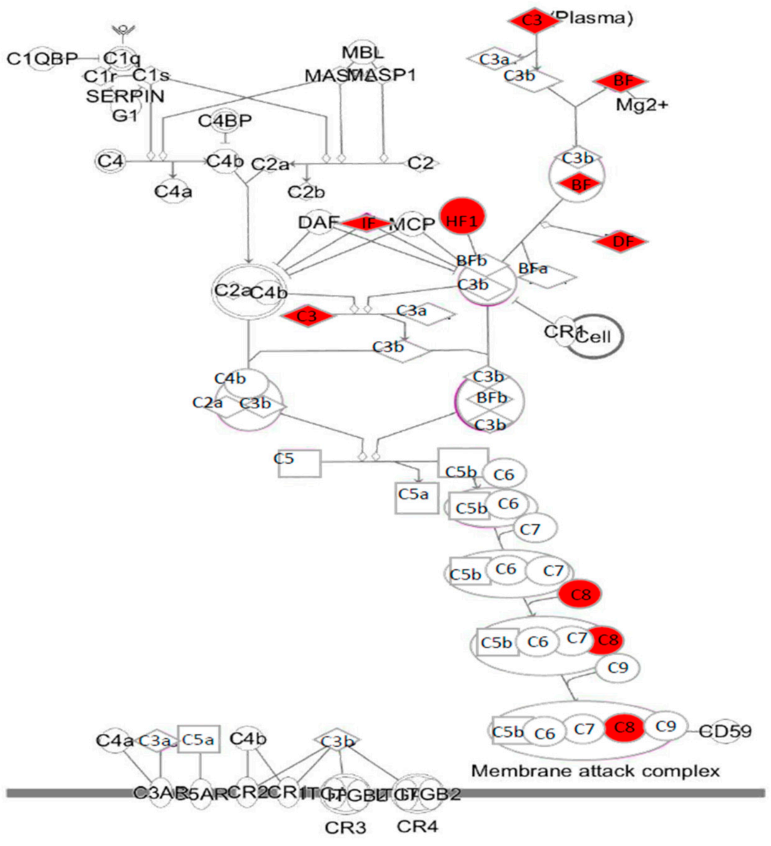

Supplementary Figure S2: Representation of the protein-protein interaction networks of complement activation. Functional enrichment analysis of the proteins identified in the HDL proteome dynamics study revealed increased turnover rates of alternative and terminal complement activation pathways (denoted in red) upon high-fat feeding in wild type mice for four weeks.

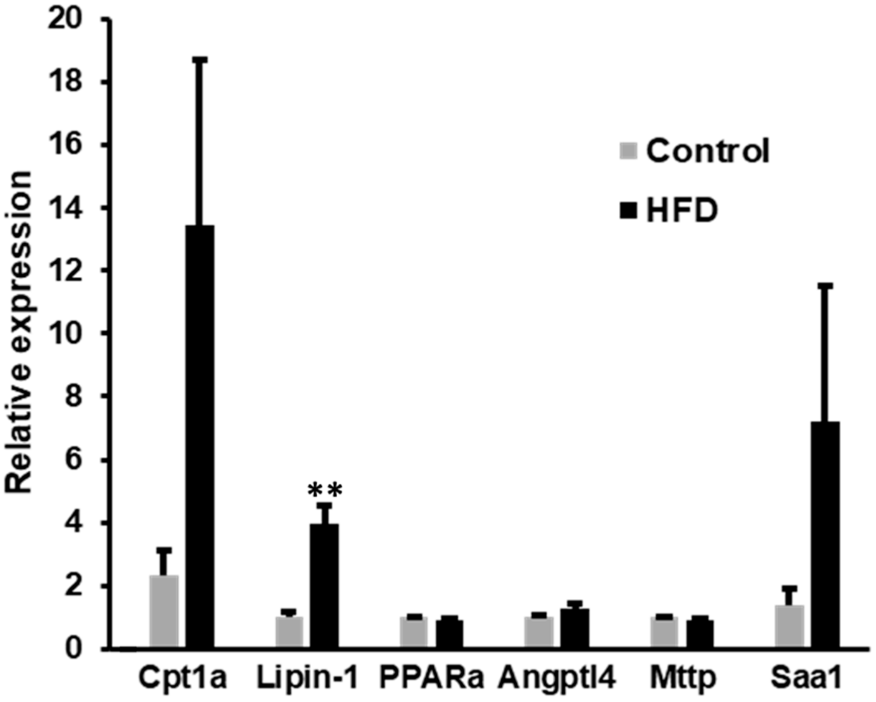

Supplementary Figure S3: Gene expression analysis using RNA isolated from livers of mice fed either the control diet or the high-fat diet. At the end of the study, the livers from the mice were harvested and RNA was isolated. Complementary DNA (cDNA) was synthesized from the RNA and the quantitative real-time polymerase chain reaction (qRT-PCR) was performed. The relative expression of indicated genes was calculated using the ΔΔCt method. Statistical significance, **indicates $P < .01$, n=6-7/group.

Supplementary Table S1: Primer sequences used for quantitative real-time polymerase chain reaction (qRT-PCR)

	Direction	Sequence
Cpt1a	forward	GGAGGTTGTCCACGAGCCAG
	reverse	TCATCAGCAACCGGCCCAAA
Lipin-1	forward	CCCTCGATTTCAACGTACCC
	reverse	GCAGCCTGTGGCAATTCA
PPARa	forward	AACATCGAGTGTCGAATATGTGG
	reverse	CCGAATAGTTCGCCGAAAGAA
Angptl4	forward	CATCCTGGGACGAGATGAACT
	reverse	TGACAAGCGTTACCACAGGC
Mttp	forward	ATACAAGCTCACGTACTCCACT
	reverse	TCTCTGTTGACCCGCATTTTC
Saa1	forward	AGTGGCAAAGACCCCAATTA
	reverse	GGCAGTCCAGGAGGTCTGTA
GAPDH	forward	CTCATGACCACAGTCCATGCCA
	reverse	GGATGACCTTGCCCACAGCCTT
